# Modular Droplet‐Based Microfluidic Platform for Functional Phenotypic Screening of Natural Killer Cells

**DOI:** 10.1002/smtd.202500236

**Published:** 2025-04-21

**Authors:** Florian Aubermann, Senne Seneca, Tomáš Hofman, Irene Garcés‐Lázaro, Karim Ajmail, Kai Daubner, Adelheid Cerwenka, Ilia Platzman, Joachim P. Spatz

**Affiliations:** ^1^ Department of Cellular Biophysics Max Planck Institute for Medical Research 69120 Heidelberg Germany; ^2^ Institute for Molecular Systems Engineering and Advanced Materials Heidelberg University 69120 Heidelberg Germany; ^3^ Max Planck School Matter to Life 69120 Heidelberg Germany; ^4^ Department of Immunobiochemistry Mannheim Institute for Innate Immunoscience (MI3) Medical Faculty Mannheim Heidelberg University 68167 Mannheim Germany; ^5^ European Center for Angioscience (ECAS) Medical Faculty Mannheim Heidelberg University 68167 Mannheim Germany

**Keywords:** droplet‐based microfluidics, natural killer cell phenotypes, negative pressure, supervised deep learning

## Abstract

Natural Killer (NK) cells, as key effector cells of the innate immune system, display high heterogeneity in their ability to kill target cells. The underlying mechanisms remain poorly understood. Here, a droplet‐based microfluidic platform is presented to identify and select NK cells with serial killing ability. To this end, primary human NK cells are encapsulated with several target cells using an efficient negative pressure‐based droplet generator. Capitalizing on the large number of possible killing events due to quantization into droplets, a convolutional neural network analysis pipeline is developed to quantify the cytotoxicity and abundance of serial killing events with high accuracy. To physically select NK cells based on their serial killing ability, MultiCell‐Sort ‐ an advanced real‐time image‐based droplet sorting module ‐ is presented. While conventional single‐cell sorters mostly evaluate intrinsically‐encoded properties, such as protein expression levels, MultiCell‐Sort can select living NK cells based on complex functional phenotypes emerging from multiple cell‐cell interactions within the droplet. This novel microfluidic phenotyping platform hereby allows the potential integration of complementary techniques to provide an understanding of regulators and markers underlying the heterogeneous nature of NK cell functional phenotypes.

## Graphical abstract

A droplet‐based microfluidic platform is developed to identify and sort Natural Killer (NK) cells with serial killing capacity. By combining high‐throughput droplet generation, deep learning‐based cytotoxicity analysis, and real‐time functional sorting, this platform enables efficient selection of highly cytotoxic NK cells. The platform enables the study of regulators and markers underlying the heterogeneous nature of NK cell functional phenotypes.

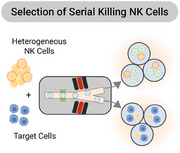

## 1 Introduction

The immune system, as a distributed organ, deploys effector cells all over the body. Many of their functions rely on cell‐cell interactions, where immune effector cells interact with various target cells to detect malignancies and pathogens.^[^ ^1^ ^]^ These cell‐cell interactions are mediated through various receptors and ligands on their surfaces. Depending on the specific set of receptors involved in the interaction, signaling cascades can evoke or inhibit the immune response.^[^ ^2^, ^3^ ^]^ Natural Killer (NK) cells are key effector cells of the innate immune system that mediate rapid cytotoxicity against infections, pathogens as well as hematologic and solid malignancies, without the need for prior activation via an antigen‐presenting cell.^[^ ^4^, ^5^ ^]^ Upon interaction with a target cell, NK cells can be activated to release cytotoxic granules leading to apoptosis of the target cell. NK cells display a heterogeneous cytotoxic potential where a subset of NK cells possess the ability to kill several target cells in a row – a process termed serial killing.^[^ ^6^, ^7^, ^8^ ^]^ Given their cytotoxic potential NK cells have gained increasing attention as cancer immunotherapy agents.^[^ ^9^ ^]^

In the bulk format of in vitro assays, it is challenging to detect and isolate distinct functional subsets of NK cells, because tracing the relevant NK cell‐target cell interactions over extended periods of time is difficult due to cellular mobility within the space. The development of single‐cell functional screening technologies has contributed to the study of such cell‐cell interactions and has generated more reproducible data. Microwell technology has improved the capability of monitoring NK cell interactions, however, the subsequent selection of specific NK cells, e.g., via fluorescence‐activated cell sorters (FACS) is experimentally challenging.^[^ ^7^, ^10^ ^]^

Droplet‐based microfluidic technology is uniquely suited to probe cell‐cell interactions, since the cell‐laden droplets create a highly controlled confinement for the interactions to take place.^[^ ^11^, ^12^, ^13^ ^]^ Moreover, the fluorescence‐activated droplet sorting (FADS) microfluidic module allows for the potential selection of relevant cells.^[^ ^14^, ^15^ ^]^ Recently, droplet‐based assays have been implemented to study NK cell‐target cell interactions. The droplet format enabled to collect precise statistics on the frequency of the serial killing phenotype.^[^ ^16^, ^17^ ^]^ In addition, the levels of cytokine expression could be quantitatively correlated to the NK cell cytotoxicity.^[^ ^18^, ^19^ ^]^

**Figure 1:**
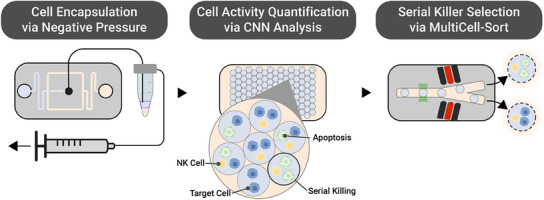
Overview of the modular microfluidic‐based platform for NK cell functional phenotyping. The encapsulation module generates cell‐laden droplets by co‐encapsulating NK and cancer cells by means of a negative pressure‐based device. In the monitoring module, the NK cell activity is screened over time and quantified via a Convolutional Neural Network (CNN) pipeline. Finally, cell‐laden droplets are sorted using an image‐based dielectrophoretic droplet sorter called MultiCell‐sort to select highly cytotoxic serial killing NK cells in the cell selection module. Afterward, the cells are released by de‐emulsification to physiological conditions for downstream assays.

Advancing on progress in the field, we developed a modular droplet‐based microfluidic platform to identify and select primary human NK cells with a serial killing phenotype. The developed platform consists of three modules: cell encapsulation, functional screening, and cell selection (**Figure** **1**). For the co‐encapsulation of primary human NK cells with K562 hematological cancer cells we designed a highly efficient and easy‐to‐implement negative pressure droplet generator. In the second module, to streamline and automate the functional screening process with high accuracy, we implemented convolutional neural network analysis. Finally, we introduce an image‐based FADS system: the MultiCell‐Sort. This third module of the platform allows for the selection of NK cells based on their cytotoxic response toward target cells. Unlike traditional FADS systems, the image‐based detection can distinguish complex scenarios such as close clusters and overlapping cells and allows to accurately distinguish killing events therefore unlocking the selection of serial killing NK cells.

The successful implementation of the developed methodology paves the way for previously difficult‐to‐achieve approaches toward cell screening for fundamental studies and clinical high‐value applications. Possible applications are cell‐based immunotherapy and numerous other biological and medical research settings, including all cell analyses, where collective cell phenomena are critical for a macroscopic read‐out (e.g., in cancer, embryonic development, wound healing, and immunology research).

## 2 Results and Discussion

### 2.1 Cell Encapsulation Using a Negative Pressure Droplet Generator

Co‐encapsulation of NK and target cells into a droplet creates individual confined spaces for each NK cell to display its cytotoxic potential. A typical droplet of 80 µm diameter contains a volume of ≈270 pl, therefore, the effective concentration of 1 cell per droplet is equivalent to a bulk concentration of 3.7 × 10^6^ cells mL^−1^. The droplet format, thus, provides a very high volumetric efficiency, i.e., 10.000 cells can be processed from 4 µL of cell suspension. Producing cell‐laden droplets from only few µL of suspension is highly challenging with a conventional setup due to high dead volumes in the tubing and reservoirs, thus making it highly challenging to impossible to use in immunological applications, where only a small number of primary NK or target cells is available. Our newly designed, easy‐to‐implement negative pressure (NP) microfluidic device allows loss‐less encapsulation of cells (Figure 1; Figure S1, Supporting Information). In the NP microfluidic device, the cell suspension (>1 µL) and the oil are directly pipetted into the inlet channel niches, and droplet formation is initiated by applying negative pressure at the outlet. Applying a negative pressure relative to the ambient atmospheric pressure at the outlet, creates a similar pressure gradient across the flow‐focusing junction as applying positive pressures in both the aqueous and oil inlets (Figure S1A, Supporting Information). The major difference is that with the negative pressure configuration, the inlets on the chip itself can be made very large and serve as reservoirs themselves of which the content is “pulled” into the microfluidic channels by the pressure gradient generated by the lower pressure in the outlet channel. Droplets leave the chip via the outlet tubing and once the reagents are fully consumed the tubing is purged by incoming air. This approach eliminates dead volumes in the reservoirs and tubing, a problem associated with conventional microfluidic setups. In conventional microfluidic setups the droplet size can be adjusted on‐the‐fly, since the inlet pressure of the continuous and the dispersed phase are controlled independently.^[^ ^20^ ^]^ During operation of the NP device the magnitude of the negative pressure is the only degree of freedom that influences both the flow rates of the dispersed and continuous phase. Consequently, even if fluctuations in negative pressure occur, the ratio of dispersed to continuous phase flow rate will remain largely unaffected, and droplet size will remain homogenous. Therefore, orientation along the milliliter scale of the syringe during pulling of the plunger is enough to ensure a reproducible production of monodisperse droplets. Confinement size has a considerable effect on the cytotoxic behavior of individual NK cells against cancer cell targets. To boost NK cell cytolytic activity toward their targets, the optimal droplet size should provide a perfect balance between the amount of space necessary for ON‐OFF conjugation as a part of the cellular recognition process and ensuring killer‐to‐target proximity. Droplets of 60 or 80 µm in diameter provide the optimized space conditions for interactions between either an NK cell and one target or an NK cell and several target cells, respectively.^[^ ^16^ ^]^ While it is not possible to control the droplet size on‐the‐fly like in a conventional setup, the droplet size can be controlled by modifying the channel geometry. A systematic way to control the droplet size is to vary the channel height (Figure S1B, Supporting Information). We produced the same channel blueprint with 30 µm channel height and 50 µm height and called these the “S‐chip” and the “M‐chip”. Each chip yield characteristic droplet sizes of 65 and 80 µm diameter, respectively (Figure S1C,D, Supporting Information). Additionally, the droplet size can be tuned by changing the serpentine lengths in the channel layout as well. This is because of the change in the hydraulic resistance ratio between aqueous and oil phase.

For convenience we integrated all components of the NP microfluidic device into a 3D‐printed 96‐well plate‐shaped frame, resulting in a portable device that can easily be adapted to microscopy and allows for cell encapsulation under sterile conditions (details are given in the Supporting Information as well as Figure S2, Supporting Information). Moreover, the use of a common laboratory syringe to generate negative pressure allows for equipment‐free fluid control. Taken together, the format of the NP encapsulation device integrates well into a cell‐focused workflow, as it allows for flexible on‐site droplet generation. In addition, due to the simplicity of the required equipment, multiple samples can be processed in parallel employing several NP devices.

### 2.2 Cell Activity Monitoring Via Convolutional Neural Network (CNN)‐Based Image Analysis

In this article we exploit droplet‐based microfluidics to develop a time‐efficient screening assay aimed at optimizing the dissection of NK cell cytotoxicity heterogeneity against cancer cells with the goal of identifying effective antitumor NK cells. Therefore, monitoring the cytotoxic activity of NK cells to determine serial killing frequencies via time‐lapse microscopy requires the screening of a large number of cell‐laden droplets. To develop the droplet cytotoxicity assay into a routine experiment, efficient and scalable analytical tools that allow for experiments with several samples being tested in parallel are required. Traditional image analysis methods can provide the necessary automation; however, droplet geometry distorts the image quality and brings cells into close proximity with each other, which complicates strategies such as cell segmentation. Therefore, we decided to train machine learning algorithms to detect killing events based on previously manually analyzed datasets. Specifically, we aimed to determine the number of living as well as dead target and effector NK cells for each droplet at each point in time. We perceived this task to be a classification problem that is solvable by training a convolutional neural network (CNN) to identify the number of cells per droplets (target, effector, etc.) directly from the raw image data. To improve the detection of cells we added several fluorescence stains: CellTrace Yellow for effector NK cells, CellTrace Violet for the target cells, and Caspase 3/7 Green activated dye for the detection of apoptosis events (Figure S3A, Supporting Information). For each sample we imaged ≈5000 droplets at 1 h intervals over a time course of 8 h.

**Figure 2:**
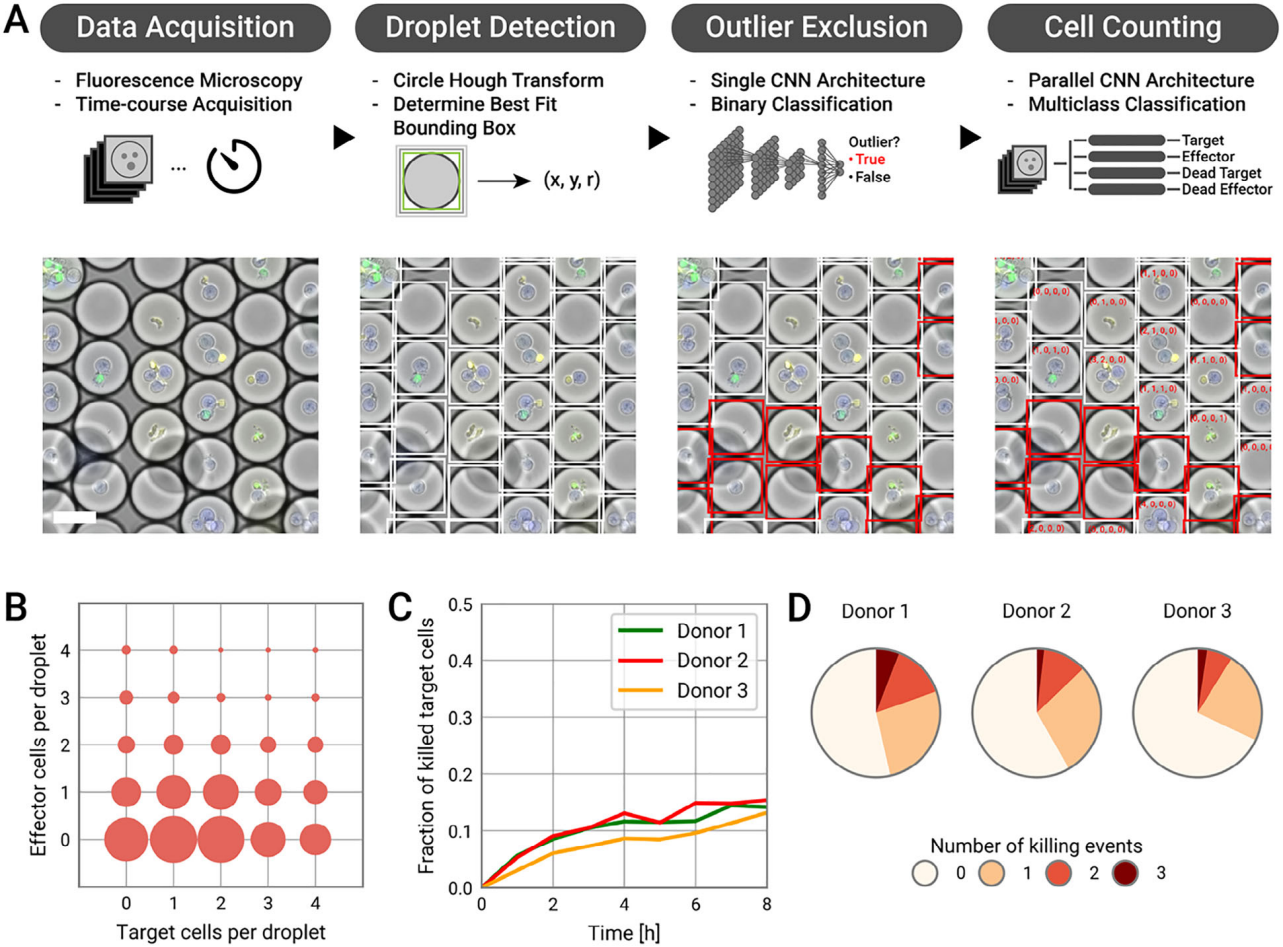
Functional screening and analysis of co‐encapsulated NK and target cells by time‐lapse fluorescence microscopy. A) Image analysis pipeline using pretrained CNN models for the prediction of droplet outliers and cell counting. Droplets are detected with the Circle Hough Transform algorithm and the optimal radius is determined for each droplet (white bounding boxes). Afterward, outlier droplets were detected using the outlier detection CNN (outliers are marked with red bounding box). Lastly, cell counts were predicted using the cell count CNN model. For each droplet the number of live and dead target and effector cells was predicted (added as text annotations). The scale bar is 50 µm. B) Co‐encapsulation distribution of Effector:Target (NK:K562) cells within the droplets C) Temporal dynamics of the fraction of dead target cells due to NK cell‐mediated killing. D) Distribution of NK cell killing events after 8 h of incubation. The analysis was restricted to droplets which contained exactly 1 NK cells and multiple target cells.

The analysis pipeline can run without user intervention and can be divided into three parts. First, droplets are detected using the Circle Hough transform algorithm.^[^ ^21^, ^22^ ^]^ For each droplet X and Y coordinates as well as the radius were estimated (**Figure** **2**A). Second, droplets with poor image quality were excluded. Such outlier droplets can be partially overlaid by an air bubble, cut off at the edge of the original image, or blurred due to a formation of a double layer of droplets in the observation chamber. To efficiently classify droplets as outliers or non‐outliers we trained a CNN model (referred to as the *outlier exclusion* model) based on the brightfield frame of each droplet to perform a binary classification (for more details see Supporting Information). For visual confirmation, outliers can be marked with a red bounding box (Figure 2A). The third, and most challenging, part of the pipeline is to extract the number of live and dead target and effector cells. We trained a more complex model consisting of four parallel CNNs (referred to as the *cell count* model), each determining one of the four parameters. During this part of the analysis brightfield and fluorescence channels are supplied to the CNN for classification. A detailed description on the network architectures as well as the training process of both the *outlier exclusion* and *cell count* model are provided in the Supporting Information.

To determine the *cell count* model's accuracy, we manually annotated the cell numbers of 1100 droplets from an experiment that was not included in the training of the model and compared the predictions to the ground truth (Figure S3B, Supporting Information). The *cell count* model reached overall accuracies of ≈90% at predicting the cell number for each cell type, meaning that cell counts in 90% of the droplets have been correctly predicted. In particular, the model performed even more accurately at predicting counts in droplets with only a few or no cells and became less accurate with increasing cell numbers. The decline in accuracy with increasing cell numbers can be attributed to two main factors. At higher cell densities, cells are more likely to overlap or form clusters, which complicates the identification of separate cells for both CNN and human annotation. Second, the impact of mispredictions is proportionally smaller at higher cell counts. For instance, if a droplet contains one cell and the CNN predicts zero or two cells, the relative error is 100%. However, for a droplet containing four cells, a misprediction of three or five cells results in a relative error of only 25%. Using the model predictions, it is possible to visualize the encapsulation stoichiometry (Figure 2B). In addition to droplets of interest that contain a suitable match of only a single NK cell together with multiple target cells, the droplets with only one NK or target cell were also used, in this case to detect the baseline viability (Figure S3D, Supporting Information). The encapsulated target K562 cells displayed a very high viability over the course of 8 h. In contrast, primary effector NK cells exhibited reduced viability after 8 h of encapsulation. Note, *cell count* viability analysis was confirmed by manually counted data. Taking NK cell viability into account, we limit the co‐encapsulation time to a maximum of 8 h to minimize the effects that the general condition of the NK cells may have on their killing efficiency.

To evaluate the NK cell‐mediated killing of target cells, we selected droplets containing a single effector NK cell and 2–4 target K562 cells, calculated the fraction of dead target cells, and extracted the baseline viability of the target cells (Figure 2C). We identified differences in cytotoxicity between different NK cell donors, highlighting the donor‐to‐donor variability of NK cells. In addition to donor‐to‐donor NK cell killing variability, heterogenous NK cell killing behavior within each population was also detected. To visualize this heterogeneity, we determined the fraction of NK cells that either killed 0, 1, 2, or 3 target cells. While the general donor‐to‐donor trend persisted, as expected, for each donor the majority of NK cells did not kill any target within 8 h of co‐encapsulation (Figure 2D).

### 2.3 Cell‐Laden Droplet Selection via MultiCell‐Sort

**Figure 3:**
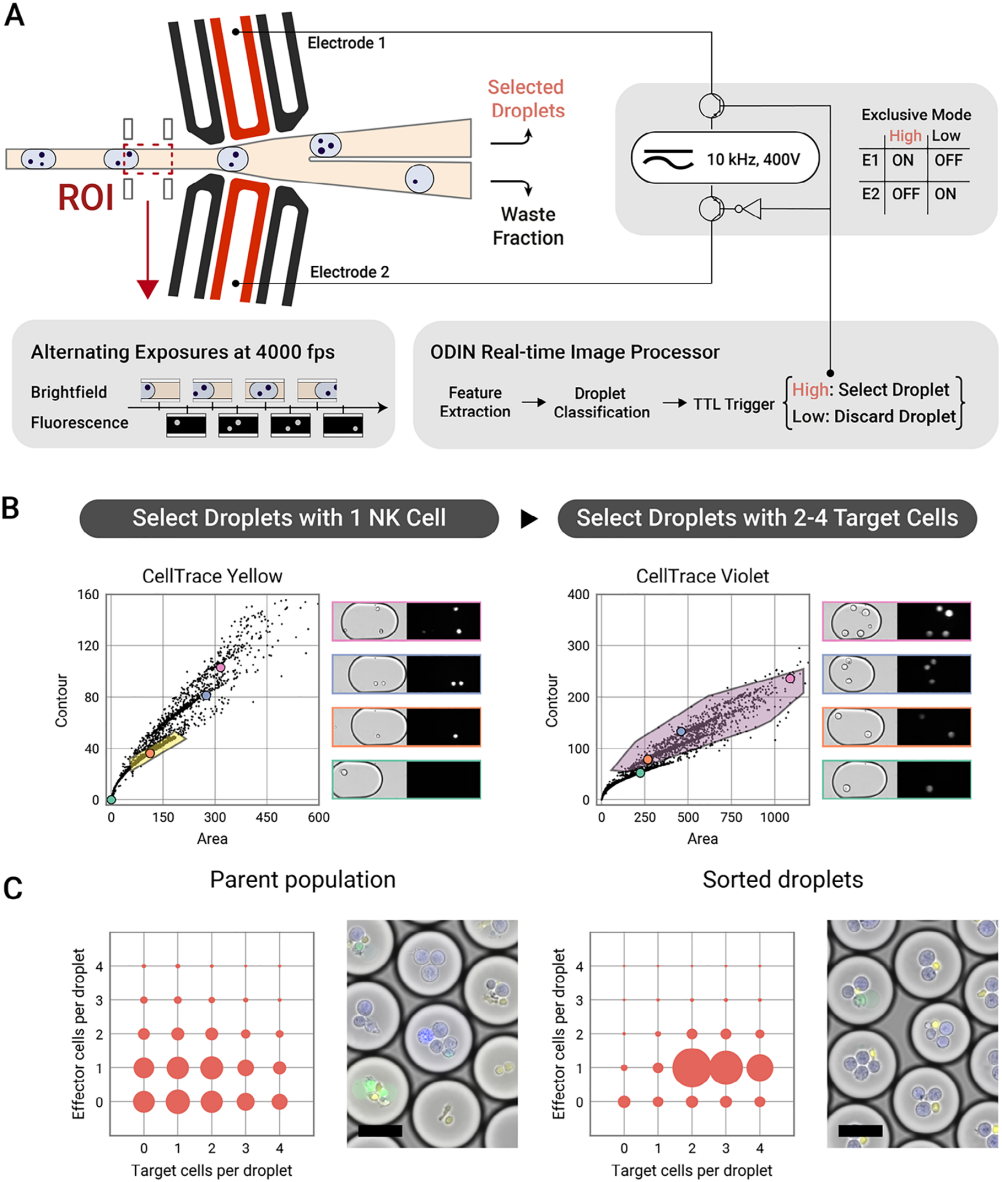
MultiCell‐Sort: Image‐based dielectrophoretic sorting of droplets using the ODIN real‐time image processor. A) Overview of the experimental setup. Droplets containing NK cells with target cells were re‐injected into a microfluidic chip featuring a Y junction. Along each arm of the Y junction electrodes can be activated which will attract passing droplets via dielectrophoretic forces. Upstream of the Y junction the ODIN real‐time image processor acquires images from a small ROI (red box) alternating the exposure between brightfield and laser‐induced fluorescence. Droplets are identified and classified, setting an output TTL trigger either to “High” if a droplet has been classified as droplet of interest or to “Low” in the opposite case. The electrodes are controlled through this TTL trigger in an “Exclusive mode”, meaning that in case of a “High” trigger the upper electrode 1 (E1) is activated and the lower electrode 2 (E2) is inactivated. In the case of a “Low” trigger, the activation pattern is inverted. B) Dot plots showing the gating strategy. During real‐time analysis of the acquired images a threshold is applied to fluorescence images resulting in binary “black‐white” images. Fluorescent cells will, thus, be represented as white circles. For each droplet the area of all circles as well as the sum of all circumference pixels is calculated (shown in the scatter plot). Droplets with either 1, 2, or 3 fluorescent cells form distinct groups. For the CellTrace Yellow channel, droplets containing exactly 1 NK cell are selected with a polygon. For the CellTrace Violet channel droplets containing 2–4 target cells are selected. Both polygons combined with a logical AND operation thus leading to a selection of droplets with 1 NK cell and 2–4 target cells. C) Microscopy analysis of the stoichiometry of the unsorted parental droplet population in comparison to the selected MultiCell‐sorted droplets. The scale bars are 50 µm.

Screening of NK cell activity revealed several functional phenotypes, ranging from NK cells that didn't kill any target cells to those that exhibited serial killing activity. Efficient high‐throughput microfluidic‐based selection and sorting of those NK cells with a serial killing functional phenotype is of a potential interest for downstream immunological assays and biomedical applications. However, achieving a high‐throughput droplet sorting requires fast data acquisition and analysis, typically within a few milliseconds. Furthermore, the droplets are in motion, which introduces motion blur and thereby reduces the data quality. Therefore, a droplet sorter will have to balance the computational complexity and speed – on one hand an advanced algorithm is necessary to accurately detect multiple cells per droplet but on the other hand, the processing must remain fast enough to enable real‐time decision making. Overcoming these technical challenges, we developed the MultiCell‐Sort module (**Figure** **3**A), which can detect and select droplets with complex and specific stoichiometry.

This novel MultiCell‐Sort platform consists of the ODIN image‐processor^[^ ^23^ ^]^ and an advanced electrode‐integrated microfluidic system. ODIN‐based image analysis of the cell‐laden droplet content has unique advantages over photomultiplier tube (PMT)‐based fluorescence detection methods. In conventional FADS systems, fluorescence signals are captured by a photomultiplier tube (PMT), and a signal trace is recorded and analyzed. A droplet containing a fluorescent cell passing through the laser beam will thus generate a peak in the signal. Depending on the amplitude of the peak or on the integrate area, an estimate about the number of cells in the droplet can be made. However, variations in cell size, stain intensity or potential overlap of cells, the resulting PMT signal can be very heterogenous (Figure S4, Supporting Information). Using an image‐based detection not only the absolute strength of the fluorescence signal, but also its spatial arrangement can be used to decode the actual number of cells present in a given droplet. This is essential for multi‐cell‐laden droplets, as two smaller cells that are close to each other are very difficult to distinguish from a single larger cell, if only the absolute strength of the fluorescence signal would be evaluated. Leveraging the image‐based detection, we selected geometric features that can clearly distinguish such scenarios. While two smaller cells might have the same area compared to a single larger cell, the two smaller cells will have a larger total contour (Figure 3B). We implemented this geometric relation by transforming the fluorescence images into binary images via thresholding. Following the transformation, the sum of all pixels (i.e., the area) was plotted against the sum of pixels on the cellular outline (i.e., the contour) to reveal separate clusters corresponding to either droplets containing 1, 2, 3, or 4 cells. Overall, this strategy yields a good separation of events but also keeps computational load low. To quantify both the number of NK cells and K562 target cells, we expanded the setup to a multi‐color acquisition, where laser lines of 561 and 405 nm wavelength are used to detect CellTrace Yellow stained NK cells and CellTrace Violet stained target cells, respectively. By gating on droplets containing ideal encapsulation stoichiometries of one NK cell and more than two target cells, the ODIN real‐time processor can classify passing droplets and send a Transistor‐Transistor Logic (TTL) signal to the function generator that controls the sorting unit (Figure 3A–C).

For droplet sorting we used two sets of electrodes that attract droplets by dielectrophoretic forces either into the selection or the waste channel (Figure 3A). Importantly, the channel and electrode design is symmetric and the sorting mechanism is not based on a hydraulic resistance bias. Instead, droplets are either actively sorted into the negative or the positive channel. To ensure a precise timing, the electrodes are activated in an exclusive mode, meaning either the upper or lower electrodes are activated depending on the TTL trigger from the ODIN processor. Although having two sets of electrodes adds complexity to the chip and hardware, it enables droplet sorting at high rates independent of the rate of positively selected droplets. Important to mention here is that hydraulic resistance bias‐based designs have limitations for high positive event rates, since the smaller positive sort channel can get choked after several droplets have been positively sorted in a row. In contrast, the symmetric design can accurately sort droplets at 100 Hz rates.

We sorted droplets with optimal encapsulation stoichiometry and determined the accuracy of the MultiCell‐Sort by analyzing the encapsulation stoichiometry using the previously described image analysis pipeline. We achieved a high purity of 76% correctly matched droplets, which marks a fourfold enrichment of the desired droplet population compared to the parental droplet population (Figure 3C). This example highlights the functionality as well as the versatility of the MultiCell‐Sort platform. In principle, the sorting criteria can be changed such that droplets with different stoichiometries are of interest.

### 2.4 Serial Killer NK Cell Selection Using MultiCell‐Sort

**Figure 4:**
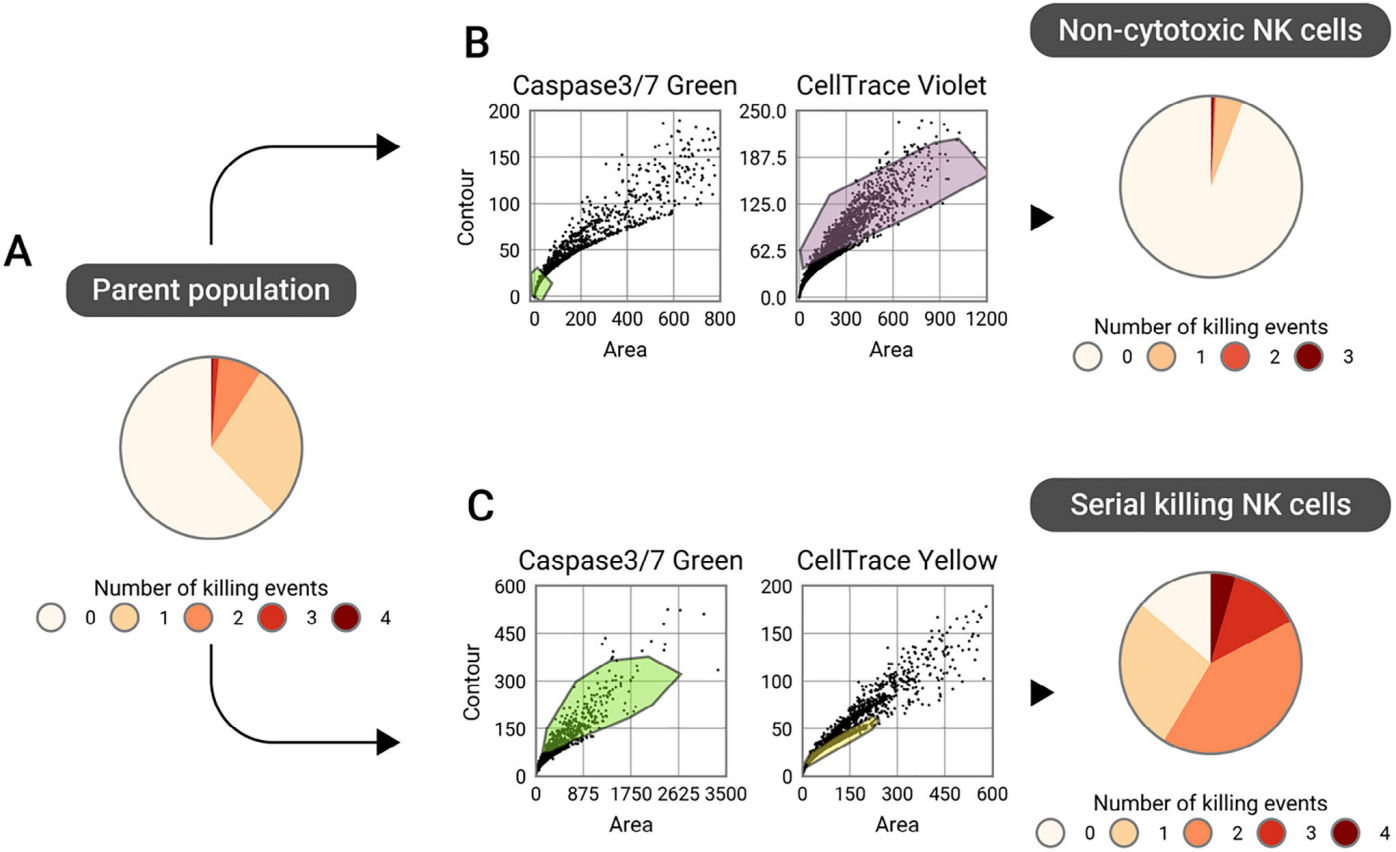
Selection of serial killer NK cells via MultiCell‐Sort. A) Distribution of killing events displayed by the unsorted parent NK cell population. B) Dot plots showing the gating strategy for the selection of droplets containing non‐cytotoxic NK cells by two sorting criteria employed during MultiCell‐Sort. First, no dead cells should be present in the droplet (Caspase 3/7 Green stain for apoptosis detection). Second, several target cells were present in the droplet. The fraction of non‐cytotoxic NK cells was evaluated via fluorescence microscopy. C) Serial killer cell selection with gating on droplets containing several dead cells as well as just a single NK cells. The fraction of serial killer cells was evaluated via fluorescence microscopy.

In the previous section we outlined how the MultiCell‐Sort module can be employed to select droplets containing specific cell type matches. Next, we demonstrate how this module can be used to select specific functional phenotypes, such as serial killing NK cells, for use in various downstream assays. Specifically, serial killing as well as non‐killing NK cells can be isolated to offer maximal resolution for subsequent use in various downstream assays. To detect dead cells, we expanded the setup with a 488 nm laser line to enable tri‐color acquisition. We co‐encapsulated NK and target cells and incubated the droplets for 6 h to allow NK cells to perform killing events. Non‐cytotoxic NK cells are cells that did not trigger a single apoptosis event within 6 h albeit being encapsulated with several target cells. Therefore, gating on droplets that contain multiple target cells and not a single dead cell during MultiCell‐Sort should yield only non‐cytotoxic NK cells (**Figure** **4**A). We performed the sort, collected the selected droplet, and analyzed them in static conditions under a fluorescence microscope. Using the CNN image analysis, we confirmed that 94% of the sorted non‐cytotoxic NK cells indeed did not kill even once. Finally, to select serial killing NK cells, we gated on droplets containing a single NK cell and two or more dead cells. Thereby we excluded scenarios of multiple NK cells in a single droplet collectively killing several target cells. By analyzing the selected droplets as before, we confirmed that ≈60% of NK cells are serial killers. Compared to the original parent population that contained ≈9% of serial killing NK cells this presents a 6.7‐fold enrichment in the serial killing NK cells. Taken together, the two resulting NK cell populations provide high contrast in their killing behavior compared to their heterogeneous parent population. To demonstrate the applicability of the MultiCell‐Sort for downstream experiments, we released the sorted NK cells from the droplets via de‐emulsification. We purified the cells by removing any remaining target cells on a FACS and confirmed that the NK cells indeed remain viable (Figure S5A, Supporting Information). Furthermore, we expanded the non‐cytotoxic and serial killing NK cells separately for 4 days and measured their growth rate (Figure S5B, Supporting Information). Interestingly, we found that serial killing NK cells proliferated faster than non‐cytotoxic NK cells, confirming that indeed functional differences between the two cell populations are present.

## 3 Conclusion

In this work we present a versatile phenotypic screening platform for the high‐throughput analysis of NK cell‐target cell interactions. We provide an efficient and scalable method of cell encapsulation utilizing a negative pressure‐based microfluidic device. Further, we implemented a streamlined CNN‐based analysis pipeline to detect and classify NK cells based on their cytotoxic potential. Expanding beyond cell monitoring, we provide means for NK cell selection via the MultiCell‐Sort module. To the best of our knowledge this is unique in its degree of flexibility to select droplets of desired cell‐cell stoichiometry and cytotoxic activity. The full potential of the droplet‐based phenotypic screening platform will be unlocked when the MultiCell‐Sort is combined with powerful downstream analysis, since selected NK cells can be recovered from the droplets via de‐emulsification. After recovery, cells can be potentially expanded and tested in preclinical and clinical settings. By conjugating single‐cell transcriptomics and mass spectrometry to the pipeline, deep fingerprints of the serial killing phenotype can be extracted. This could lead to the potential discovery of novel markers, signatures, and mechanisms associated with serial killing. Moreover, the platform is widely applicable to several immune effector cells as well as target cells and combinations thereof and can be tailored for other readouts of immune cell activation and killing capacity including cytokine production. Especially in the context of tumor‐resistance, the phenotype of the target cells – and not just of the immune cells – is highly relevant. Overall, the wide adaptability of the phenotypic screening platform promises to aid the development of more potent immunotherapies.

## 4 Experimental Section

### Microfluidic Chip Production

Microfluidic chips were produced using standard poly‐dimethylsiloxane (PDMS)‐based soft lithography. In brief, PDMS slabs (Sylgard 184, Dow Corning, USA) were cured on silicon master wafers. The channel layouts on the master wafer are shown in Figure S1 (Supporting Information). In‐ and outlets were punched using 0.75 mm biopsy punchers. Inlets for negative pressure chips were punched with a 3 mm biopsy puncher. The PDMS slabs were cleaned using 70% ethanol and pressurized air and bonded onto glass slides using low‐pressure oxygen plasma (200 W, 30s, Tepla 300 Semi‐Auto Plasma Processor, PVA TePla, Germany). Electrodes were added to the chip by pushing a low‐melting point Indium alloy (In_0.51_Bi_0.325_Sn_0.165_, Indium Corporation of America, USA) into the channel, while the chip was resting on a hot plate (75–85 °C) to facilitate melting.

### Cell Culture

K562 cells (Cell Line Service, Germany) were cultured in RMPI (Gibco Glutamax, 10% FCS, 1% Penicillin‐Streptomycin, Thermo Fisher Scientific, Germany) at 37 °C (humidified atmosphere, 5% CO2). Cells were passaged every 2–3 days to maintain a concentration of 200k‐800k cells mL^−1^. For the preparation of primary NK cells, peripheral blood mononuclear cells (PBMCs) were first isolated from healthy donors’ blood buffy coats (provided by DRK‐Blutspendedienst Baden‐Württemberg‐Hessen (Mannheim, Germany)) by density centrifugation using Pancoll (PAN Biotech). Primary human NK cells were then purified from PBMCs using the Human NK Cell Isolation Kit (Miltenyi Biotec), reaching ≈95% purity. NK cells were cultured at a concentration of 2 × 10^6^ cells mL^−1^ in NK MACS media (Miltenyi Biotec, Germany) supplemented with 10% human AB serum, 1% Penicillin/Streptavidin, 2 nm L‐Glutamine and 400 U mL^−1^ of IL‐2 (Miltenyi Biotec, Germany or TECIN (Teceleukin) provided by the National Cancer Institute). Written informed consent from the blood donors was obtained and ethical approval 87/04 was granted by the Ethik Kommission II of the Medical Faculty Mannheim (Mannheim, Germany).

### Staining Protocols and Cell Preparation

NK cells and target cells were stained with CellTrace Yellow and CellTrace Violet, respectively (Thermo Fisher Scientific, Germany). Both dyes were prepared in PBS to a final concentration of 2.5 µm, according to the manufacturer's protocol. For encapsulation, cells were resuspended to an appropriate concentration in encapsulation medium. Encapsulation medium was prepared as RPMI (no Phenol red, 2% FCS) with Incucyte Caspase 3/7 Green (10 µM final concentration, Sartorius, Germany). To achieve cell encapsulation with an average effector cell:target cell ratio of 1:2, NK cells and target cells were resuspended to a final concentration of 3 × 10^6^ and 6×10^6^ cells mL^−1^, respectively.

### Microfluidic Experiments

In all microfluidic experiments 5 w/w % RAN‐008 surfactant in HFE7500 (RAN Biotechnologies, USA) was used as the continuous phase. The Eppendorf tubes were connected to the microfluidic chip using 1/32″ OD PTFE tubing (Darwin Microfluidics, France). Cell encapsulation via negative pressure was conducted using the chip depicted in Figure S6A (Supporting Information). A high‐speed camera recording of the encapsulation process is provided in Video S1 (Supporting Information). A detailed description of the procedure is given in Supporting Information.

Droplet sorting experiments were performed using the chip depicted in Figure S6B (Supporting Information) and an OB1 MK3+ pressure controller setup (Elveflow, France). Droplets were reinjected at pressures of ≈100–120 mbar. Spacing oil was injected at 300–400 mbar. Droplets were imaged at 4000 fps in the detection ROI by the ODIN sensor (Sensific, Germany). To detect fluorescence signals, lasers of 405, 488, and 561 nm were used for excitation with an excitation time of 250 µs (Oxxius, France). To generate binary images, thresholds were manually adjusted. An alternating electric potential was applied to the electrodes to sort droplets. A function generator (HM8150, Rhode & Schwarz) generated a 4 V (p‐p) sinus waveform which was then amplified x100 using a high‐voltage amplifier (Model 2210, Trek). TTL signals to switch the electrodes were generated by the ODIN sensor. A detailed description of the ODIN operation could be found in.^[^ ^23^ ^]^ The sorting accuracy was monitored with an external high speed camera (Phantom v7.2511, Vision Research, USA). To ensure precise droplet selection, the trigger delay (2ms) as well as trigger length (8–10 ms) was adjusted as needed to synchronize the electrode switching at the droplet junction with the droplet flow rate. A high‐speed camera recording of the droplet sorting process is provided in Video S2 (Supporting Information). The overall accuracy of the droplet sorting was best measured by collecting the sorted droplets and analyzing them via fluorescence microscopy under static conditions. Thereby, all possible sorts of errors originating from various steps in the experiment were integrated. To release cells from droplets, the emulsion was broken by the addition of 1H, 1H, 2H,2H‐perfluor‐1‐octanol (20 v/v % in HFE7500, Sigma Aldrich, Germany) while PBS was added to the emulsion to resuspend the released cells. Each experiment was conducted as biological triplicates.

### Time‐Lapse Microscopy

To monitor encapsulated cells over time, droplets were injected into an observation chamber (µ‐Slide VI 0.4 µm, Ibidi GmbH, Germany) and imaged with an inverted fluorescence microscope (DMi8, Leica Microsystems, Germany). Images were acquired at 16 bits with a 10x objective and tile scans were stitched together using the LAS X Navigator (Leica Microsystems, Germany).

### Image Analysis Pipeline

A detailed description of the image analysis pipeline is given in the Supporting Material. In brief, the image analysis pipeline was implemented in Python programming language (Version 3.12). Major steps were the droplet detection, outlier detection, and the *cell count* prediction. Once cell count data was available, different statistics could be extracted from the dataset.

## Conflict of Interest

The authors declare no conflict of interest.

## Author Contributions

F.A., I.P., and J.P.S. conceived the research and conceptualized the microfluidic platform. F.A., K.A., and K.D. trained the CNN models. T.H. and I.G.L provided primary NK cells. The paper was written by F.A., S.S., I.P., and J.P.S. with contributions from all of the coauthors. A.C., I.P., and J.P.S. acquired resources. All authors agreed to the final version of the manuscript.

## Supporting information



Supporting Information

Supplemental Video1

Supplemental Video2

## Data Availability

The data that support the findings of this study are available from the corresponding author upon reasonable request.
