# Supplementary material for: Modular Droplet‐Based Microfluidic Platform for Functional Phenotypic Screening of Natural Killer Cells
Source: Small Methods. 2025 Apr 21;9(8):2500236. doi: 10.1002/smtd.202500236 (PMC12391620; doi:10.1002/smtd.202500236)
Supplement: Supplementary file 1 — Supporting Information [file SMTD-9-2500236-s002.docx]

**Modular Droplet-based Microfluidic Platform for Functional Phenotypic Screening of Natural Killer Cells**

Florian Aubermann, Senne Seneca, Tomáš Hofman, Irene Garcés-Lázaro, Karim Ajmail, Kai Daubner, Adelheid Cerwenka, Ilia Platzman^*^ and Joachim P. Spatz^*^

F. Aubermann, S. Seneca, K. Ajmail, K. Daubner, I. Platzman and J. Spatz:

Department of Cellular Biophysics, Max Planck Institute for Medical Research, Heidelberg, Germany.

F. Aubermann, S. Seneca, K. Ajmail, K. Daubner, I. Platzman and J. Spatz:

Institute for Molecular Systems Engineering and Advanced Materials, Heidelberg University, Heidelberg, Germany.

F. Aubermann, K. Ajmail and J. Spatz:

Max Planck School Matter to Life, Heidelberg, Germany.

T. Hofman, I. Garcés-Lázaro and A. Cerwenka:

Department of Immunobiochemistry, Mannheim Institute for Innate Immunoscience (MI3), Medical Faculty Mannheim, Heidelberg University, Mannheim, Germany.

A. Cerwenka:

European Center for Angioscience (ECAS), Medical Faculty Mannheim, Heidelberg University, Mannheim, Germany.

Corresponding authors: ilia.platzman@mr.mpg.de; spatz@mr.mpg.de


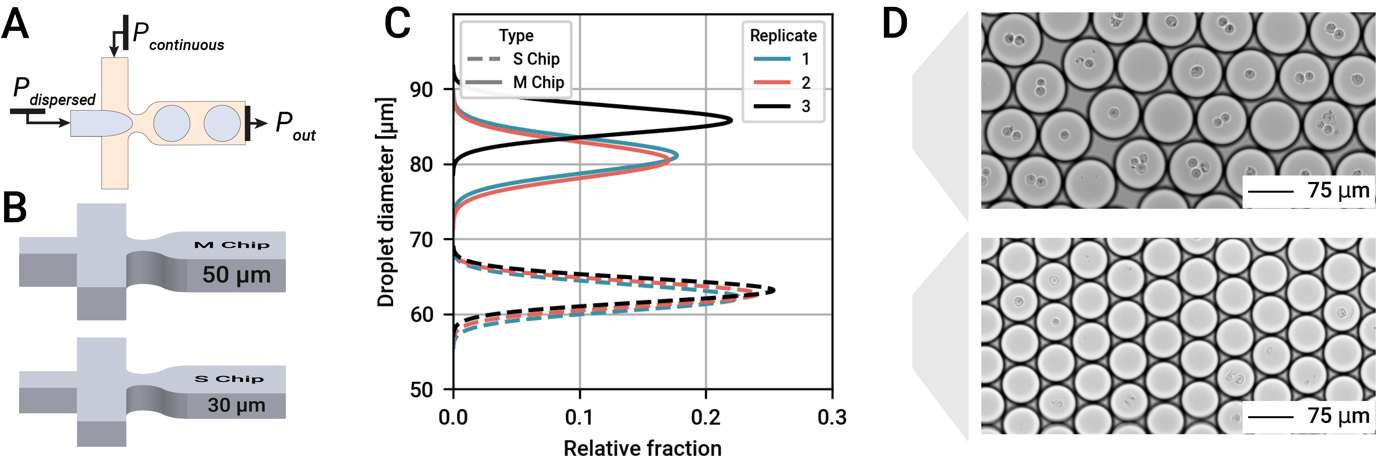


Figure S1: Droplet size control as part of the negative pressure cell encapsulation. A: During droplet formation the droplet size is determined by three pressures: the pressure of the dispersed phase (i.e., the cell suspension), the pressure of the continuous phase (i.e., the oil phase) and the pressure at the outlet. Applying negative pressure to the outlet creates a pressure gradient throughout the device that drives the fluids through the channels. B: The negative pressure chip was produced in two sizes: the S-chip with 30 µm and the M-chip with 50 µm channel height. C&D: S- and M-chips produce droplets of characteristic sizes of around 65 µm and 80 µm, respectively.


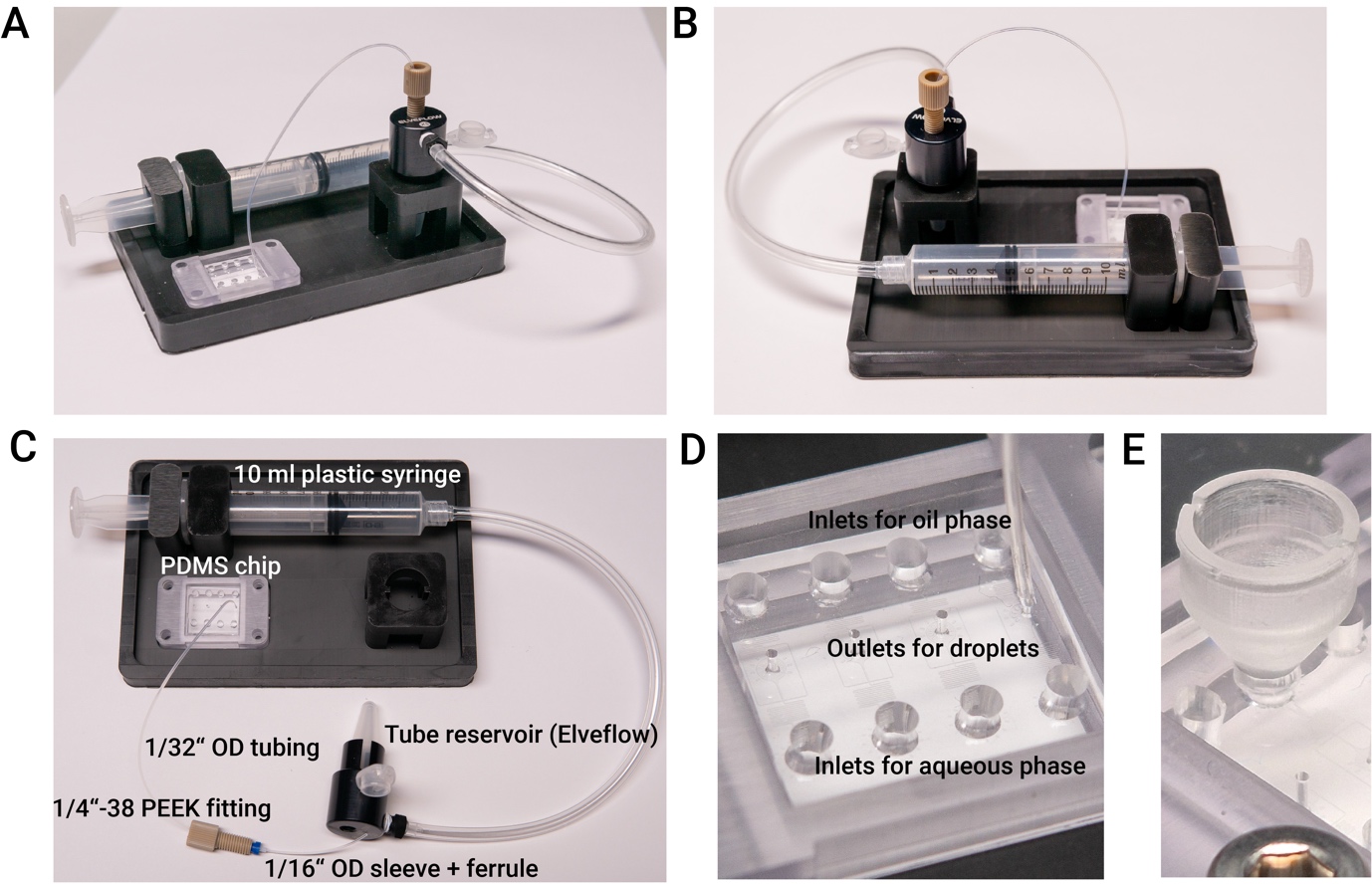


Figure S2: Negative pressure microfluidic device for efficient cell encapsulation. A&B: Side views of the 3D printed frame. C: All parts that are required for operation of the negative pressure device are depicted. D: Close-up view of the PDMS chip (M-chip). E: If larger amounts of cell suspension are processed, 3D-printed reservoir extenders can be inserted into the inlet niches.


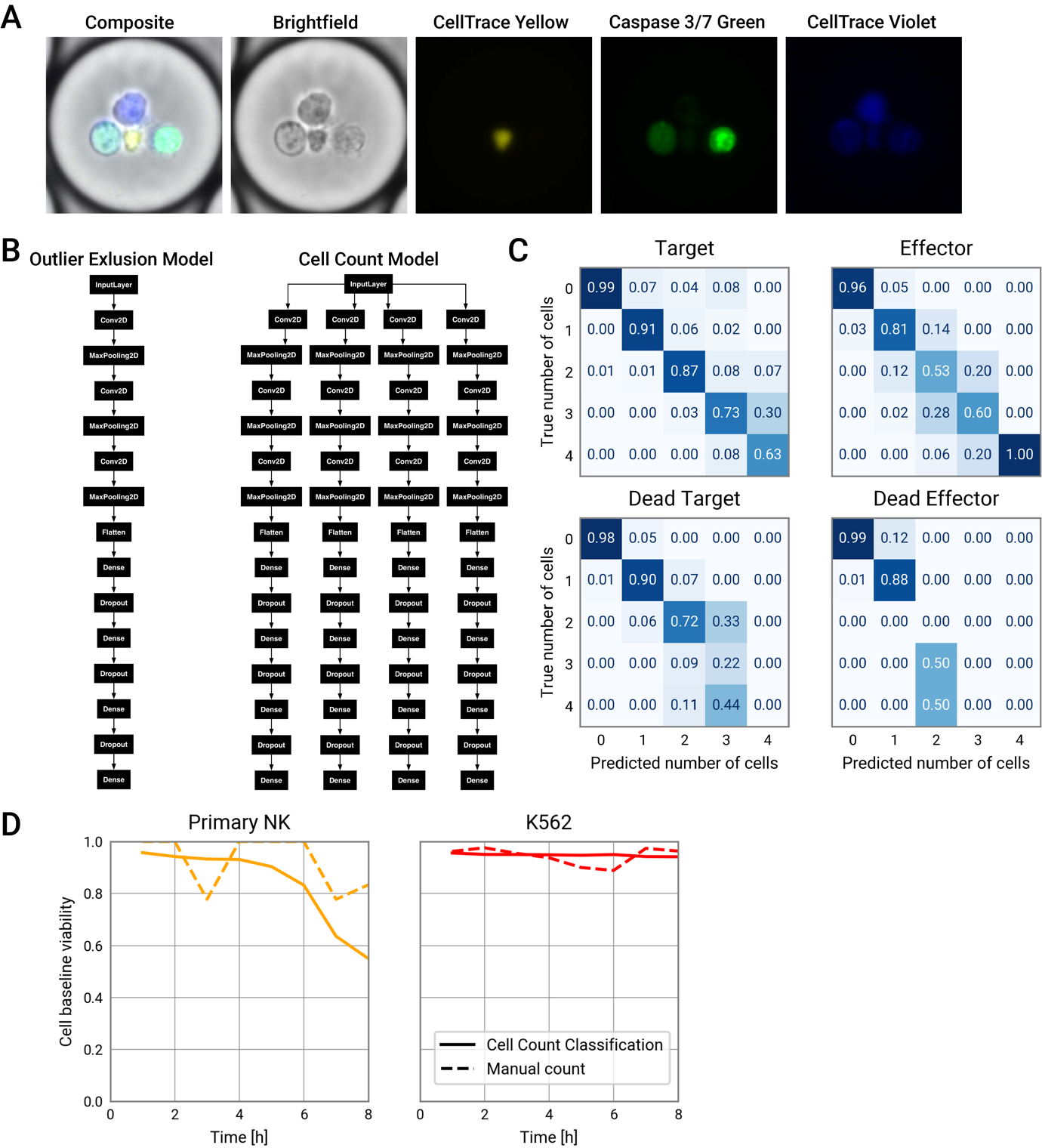


Figure S3: A: Representative fluorescence and bright-field images of the cell-laden droplets. NK cells were stained with CellTrace Yellow; target cells were stained with CellTrace Violet. A Caspase3/7 Green activated dye was added indicating apoptotic cell death. B: CNN model architectures for the outlier exclusion and cell count model. C: Confusion matrices for the cell count model. D: Baseline viability of primary NK cells (effector cells), K562 (target cells). The baseline viability was determined from manually counted data using droplets that exclusively contained only the respective cell type.


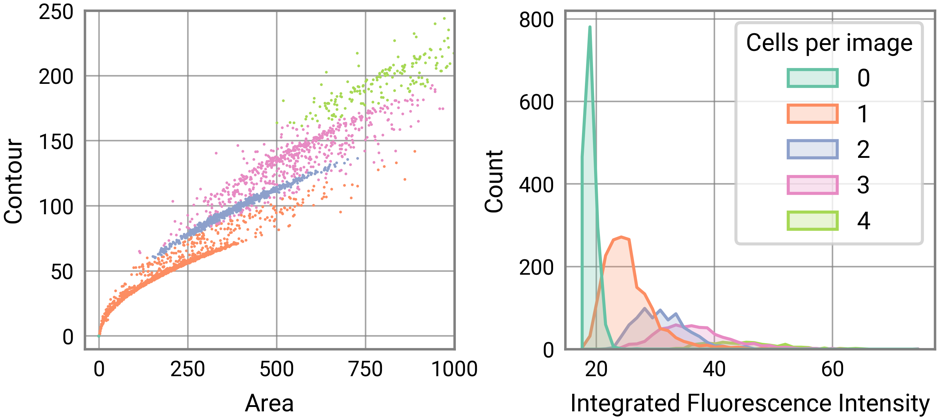


Figure S4: Image-based detection of fluorescent cells provides higher information content about the cell number compared to an integrated fluorescence signal. In the image-based analysis the area as well as the contour of the cells’ fluorescence signal can be quantified, yielding a good differentiation between images containing none, one, two, or more fluorescent cells. In contrast, when the fluorescence signal is integrated the spatial information is lost and the distinction between the number of fluorescent cells per image is blurred.


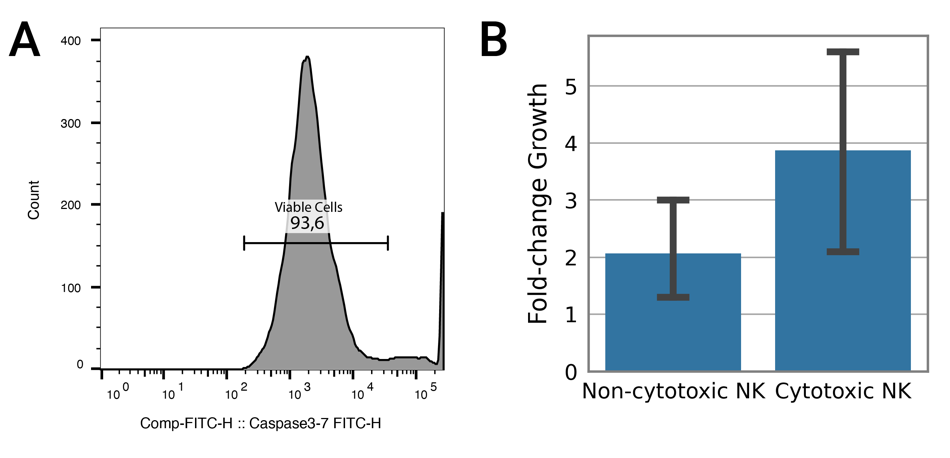


Figure S5: Downstream viability of sorted NK cells. Following MultiCell-Sort, NK cells were released from the droplets and purified by FACS to remove dead target cells. Among the NK cells, viability was assessed (A). Non cytotoxic and cytotoxic NK cells were expanded separately for 4 days and their growth rate was measured (B). The experiment was conducted with n=3 biological replicates.


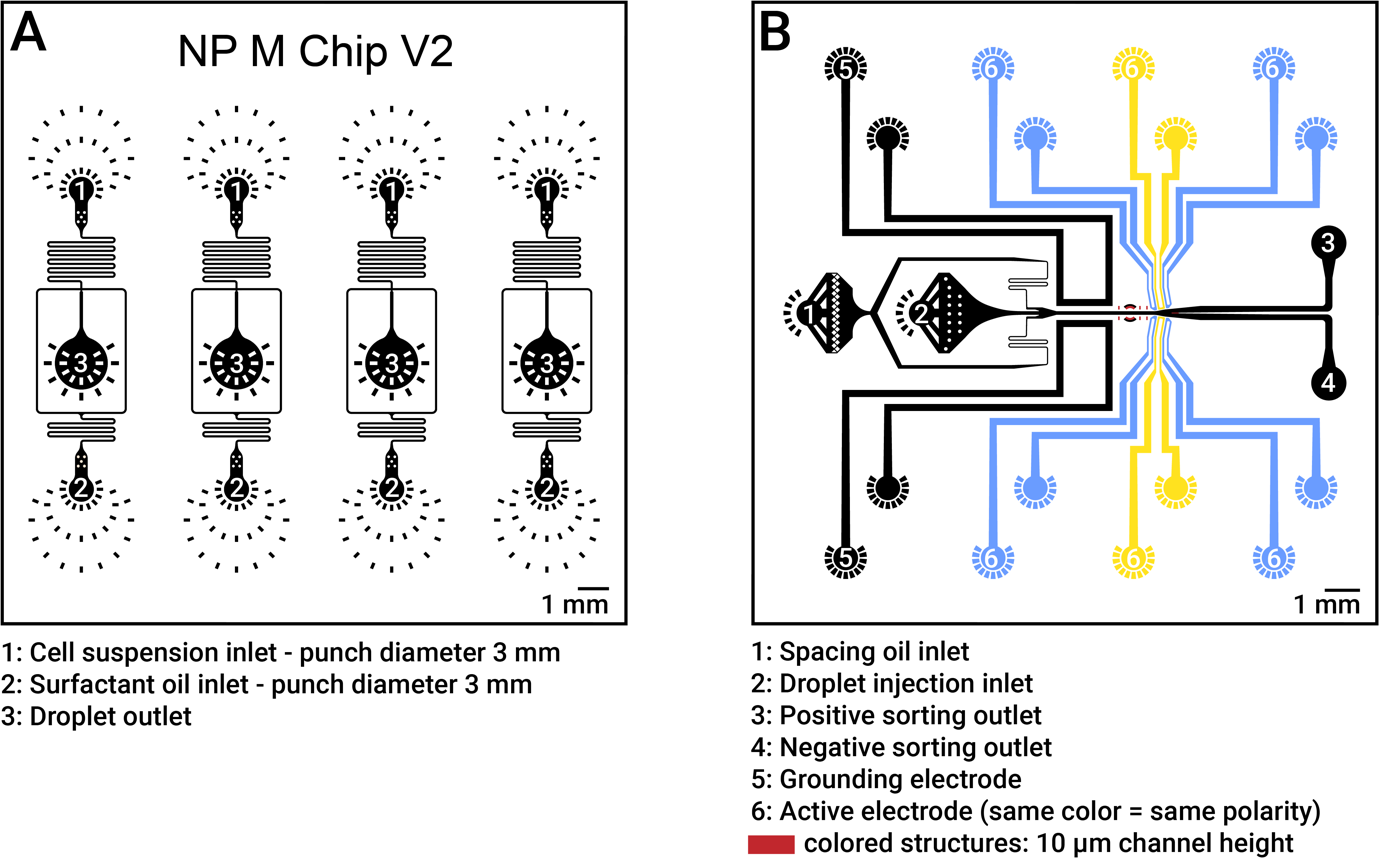


Figure S6: Blueprints of the microfluidic devices used. A: Blueprint of the droplet production chip. If a chip was designed for negative pressure-based droplet production, the outer circles at the inlet were used as a guide for punching inlets with a 3 mm diameter puncher. In the case of conventional positive pressure chips all inlets were punched with 0.75 mm. B: Blueprint of the sorting chip. All in- and outlets were punched with a 0.75 mm puncher.

## Video S1:

High-speed camera recording of the cell encapsulation. Primary NK cells were co-encapsulated with K562 target cells.

## Video S2:

High-speed camera recording of the droplet sorting. The white box marks the detection ROI of the ODIN sensor. Droplets of interest (*i.c.* droplets containing serial killing NK cells) are sorted into the upper channel which is indicated by the switching of the electrodes. The recording mostly shows brightfield signals. The much dimmer fluorescence signals occasionally can be observed as a white signal in the detection ROI.

## Supporting information – Step-by-step guide for the operation of the NP device

The inlets of the NP device can hold up to 20 µl of cell suspension (depending on the height of the PDMS block). If larger amounts of cell suspension should be processed, a 3D-printed reservoir extender can be inserted (Figure S2 E). The reservoir extender does not influence droplet production, while offering a larger load capacity of up to 200 µl of cell suspension. Once the NP device is setup according to Figure S2C, the following step-by-step guide illustrates the workflow:

- Step 1: Prior to closing the reservoir, adjust the syringe plunger to the starting position (5 mL in case of a 10 mL syringe) and tighten all connections.
- Step 2: Attach the PTFE tubing to the outlet of the microfluidic device. If necessary, use tweezers. Make sure that the tubing is inserted at least 2 mm. To reduce dead volume, the tubing should be inserted a far as possible without breaking the glass.
- Step 3: Load the aqueous phase into the water inlet. Make sure to pipette directly on to the glass bottom to avoid the formation of air pockets below the solution. Similarly, load the oil phase into the oil inlet.
- Step 4: Pull the plunger to the vacuum position. Depending on lighting conditions, it is possible to observe droplets moving up the outlet tubing. A decrease in traveling speed of the droplet phase is an indicator for vacuum leakage. In the case of vacuum leakage: open the reservoir to release the vacuum, adjust the syringe to its starting position, close the reservoir tightly, and pull the plunger again.
- Step 5: Once the water or oil reservoir is empty, air will be pulled into the outlet tubing. The remaining droplets are cleared from the tubing and collected in the Eppendorf tube. As soon as all droplets have been cleared from the tubing, open the reservoir to release the vacuum.

## Supporting information – Details concerning the image analysis pipeline

## Preparations and droplet detection

The code for the image analysis pipeline as well as an exemplary analysis is available at https://github.com/fauberma/droplet-phenotyping. The pipeline was designed to handle each experiment as a separate dataset with a unique experiment ID (expID). In addition, all relevant meta data are added to the pipeline through an excel file called ‘setup.xlsx’. This file contains relevant information on raw data location, experimental conditions, etc.

The first step is the droplet detection via the Circle Hough Transform (CHT). In order to use the CHT, a binary image of the droplet outline needs to be generated. Typically, the droplet outline has very strong contrast, allowing for robust thresholding. If the default binarization does not yield satisfying results, it might need to be adjusted. After CHT has been applied, coordinates of droplets (x,y,r), as well as information about the sample conditions are stored in a csv file ‘droplets.csv’. To make the droplets quickly accessible for downstream analysis, a database consisting of Tensorflow Record (TFRecord) files is generated, where each droplet is stored in a 128 pixel x128 pixel x number of channels frame.

## Training the outlier detection model

The outlier detection model architecture consists of convolutional as well as fully connected layers. The input layer accepts 128x128x1 arrays (brightfield channel) which are passed through three convolutional layers, each followed by a max-pool operation. After conversion to a dense layer, the results are passed through three fully connected layers, each followed by a dropout layer. Finally, the output layer consists of two nodes, representing the probability of a droplet being an outlier or non-outlier. To generate training data for this model, we divided droplets into several distinct groups by using unsupervised clustering. The motivation behind this was to possibly identify clusters that almost exclusively contain outliers, thus removing the necessity to manually identity and label droplets. More specifically, we performed K-means clustering on the 8-bit histograms of the brightfield channels of the droplets. By varying ‘k’ (the expected number of means) the dataset got divided according to the learned features of K-means. By visually studying the composition of the proposed clusters, it was possible to identify clusters containing overlapping droplets, clusters containing droplets with overlaying air bubbles as well as clusters with non-outlier droplets. Importantly, this clustering needs to be performed on each dataset separately. Otherwise, the clustering is heavily influenced by batch effects between different experiments. In summary, we could quickly annotate a large number of droplets by manually identifying optimal K-Means clustering. To train the model, we used ca. 1.000.000 droplets originating from 11 independent experiments to prevent overfitting on one particular dataset. The code for the outlier exclusion model training can be found in the ‘outlier_exclusion.ipynb’ notebook.

## Training of the cell count model

The *cell count* model consists of four parallel CNNs where each CNN has an architecture similar to the *outlier exclusion* model. The motivation was that each CNN should learn to predict one of the four categories (number of target cells, effector cells, dead target cells and dead effector cells). The input layer of the cell count model accepts 128x128x4 arrays (channel order: brightfield, yellow (effector cell stain), green (apoptosis stain), and blue (nuclear stain)). The output layer of each CNN contains five nodes, representing the probability of the droplet having 0, 1, 2, 3 or 4+ cells. To generate training data we labeled the droplet content manually by using a custom web application (can be started via wp_annotation.py). Droplets that were of poor image quality were labeled with a ‘10’ during annotation and filtered out for model training. The cell count model was trained using approximately 3500 annotated droplets from 4 independent experiments. The code of the training can be found in the ‘cell_count_v3.ipynb’ notebook.

## Outlier exclusion and cell count with pretrained models

Once the training of the CNN models is concluded, the pipeline can be used for normal analysis. In principle, after setting up the ‘setup.xlsx’ file, no additional user intervention is required.
